# Qualitative surface roughness of lithium disilicate endo-crown for pulpotomized primary molars

**DOI:** 10.1038/s41598-024-68689-w

**Published:** 2024-08-11

**Authors:** Shaimaa M. Mahfouz Omer, Shaimaa S. El-Desouky, Rania El-Saady Badawy, Shimaa M. Hadwa, Reham M. Ali Abdel Latif

**Affiliations:** 1 Pediatric Dentistry, Preventive Dentistry, and Dental Public Health Department, Faculty of Dentistry, Suez University, Suez, Egypt; 2 Pediatric Dentistry, Oral Health and Preventive Dentistry Department, Faculty of Dentistry, Tanta University, Tanta, Egypt; 3 Dental Biomaterials Department, Faculty of Dentistry, Suez Canal University, Ismailia, Egypt; 4 Pediatric Dentistry, Preventive Dentistry, and Dental Public Health Department, Faculty of Dentistry, Suez Canal University, Ismailia, Egypt

**Keywords:** Primary molars, Stainless-steel, Zirconia, Fiberglass, Lithium disilicate endo-crown, Surface roughness, Health care, Materials science

## Abstract

Rehabilitation of pulpotomized primary molars with an appropriate restoration is essential for recovering function and safeguarding the durability of the treatment. This study aimed to assess and compare the surface roughness of stainless steel (ST) crowns, zirconia (ZR) crowns, fiberglass (FG) crowns, and lithium disilicate (LD) endo-crowns as a restoration for pulpotomized primary molars also, evaluating the surface roughness of their antagonists. Sixty pulpotomized primary mandibular first molars were used for qualitative surface roughness evaluation and divided into four groups (*n* = 15/group) according to the crown type (group-ST, group-ZR, group-FG, group-LD). While the other sixty sound, unprepared primary maxillary first molars were used for evaluation of their surface roughness against the tested crowns. Specimens’ preparation and cementation were carried out according to each crown type and manufacturer’s instructions. The surface roughness was done using a two-body wear test. The data were statistically analyzed. All tested crowns showed an increased change in surface roughness, except group-ZR, which had the least change in surface roughness after mechanical wear with no statistically significant difference(P = 0.681). All crown types significantly increased the surface roughness of their antagonists after mechanical wear, except group-ST which showed insignificant affection (p ≥ 0.05). Zirconia crowns and lithium disilicate endo-crowns had the least change in surface roughness compared to other groups while SSCs showed the least tooth loss in the antagonist enamel.

## Introduction

Restoration of pulpotomized primary molars is a great challenge in pediatric dentistry due to their similar characteristics of fracture-susceptibility to the endodontically treated teeth^1^. Thus, the full coverage restorative dental material should replace the lost tooth structure with a perfect coronal seal, good esthetics, and improved mechanical &functional properties^2^.

Stainless-steel crown (SSC) is the benchmark in restoring grossly decayed primary molars; it is mainly recommended in teeth with multi-surface restorations due to developmental defects or caries, and pulp-treated teeth^3^. Stainless steel is alloys of iron and carbon that include chromium, nickel, and manganese; it is strong, resilient, and malleable. SSCs have a metal thickness of 0.2 mm and do not need extra thickness to achieve clinical acceptance^4^. SSC has superior clinical performance due to its durability, maintenance of the morphologic tooth form, and minimal technique sensitivity during placement. Despite its favorable characteristics, the metallic appearance of SSC does not fit the parents’ desires for restoring their children’s carious teeth also, expanding the necessity for alternative esthetic crowns^5^.

Esthetic restoration of primary teeth is very challenging due to increased pulp proximity to the tooth surface, small bonding surface area, high-cost treatment, and difficult child management^6^. Prefabricated zirconia crowns (PZCs) are produced and presented as an esthetic, biocompatible alternative for SSC in restoring grossly decayed primary molars. Zirconia has superior mechanical properties as it has high flexural strength, high fracture toughness, and greater strength characteristics allowing it to endure chewing forces without fracture^7^. Also, zirconia is resistant to wear and abrasion since it is relatively hard and conserves the integrity of the restoration and opposing natural teeth, maintaining a stable occlusal relationship^7^. However, its high cost and difficult crown trimming or even altering its shape are the main disadvantages of zirconia restorations^8^. Many brands have since evolved in the manufacture of zirconia crowns; however, all brands needed a significantly higher tooth reduction compared to SSCs, resulting in more time needed for crown preparation and insertion, which is an essential factor in dealing with children^9^.

Fiberglass crowns were introduced as an esthetic full coronal restoration for primary teeth, with a similar structure to fiberglass dental posts^10^. It is composed of fiberglass, aramid, carbon, and quartz filaments embedded in a composite resin substance^11^. With resin and fillers accounting for the bulk, it includes about 25 and 85% fibers, with 30 to 70% more desirable. The quantity of fiberglass mesh sheets varies from one to three in a layer which may be increased till the total thickness renders unnecessary. These sheets have approximately 0.0889 mm thickness and nearly 8.1 g/mm^2^ weight^11^. This merged structure of Figaro crowns presented superior strength, a greater degree of flexibility, and biocompatibility^12^ which are much closer to the tooth structure^13^. Moreover, they have 2.5 times greater compressive strength than stainless steel and Zirconia crowns in ball bearing and compression tests^14^. Additionally, the manufacturer claimed that Figaro crowns are white crowns that are very similar to SSCs in preparation and that they are based on flex fit technology, which allows the crowns to be placed with minimal tooth reduction saving time and providing tight margins similar to SSCs^10^.

Recently, endo-crowns have been recognized as an esthetic minimally invasive restoration for primary teeth^15^. Several various materials are suggested for the construction of endo-crowns including lithium disilicate glass–ceramic, zirconia-reinforced lithium silicate glass–ceramic, zirconia, hybrid composite resin, and computer‑aided design/computer‑aided manufacturing all‑ceramic blocks^16^. The endo-crown's performance and mechanical characteristics can be affected by the material selection^17^. It is strongly advised to utilize lithium disilicate glass–ceramic due to its good mechanical qualities, aesthetically pleasing results, and capacity to bond to tooth structure^17^. The zirconia particles that are added to the matrix of zirconia-reinforced lithium silicate glass–ceramic, improve its mechanical and physical characteristics but also weaken the link between the restoration and the tooth structure^17,18^. Because of its superior mechanical properties, zirconia is frequently utilized in high-stress circumstances like bruxism however it cannot be etched with conventional techniques, which might lead to low bond strength, possibly debonding of the restoration, and a high rate of catastrophic failures^18^.

Endo-crown is a one-piece construction integrating the post and core, to produce a full-block prosthesis. Endo-crowns are made up of a butt plane and retainer which are firmly fixed into the interior of the pulp chamber and the cavity margins^19^. This design takes advantage of the pulp cavity's macroscopic and microscopic mechanical retention^20^. Endo-crowns are particularly recommended for molars with short or weak roots, as well as in situations of a significant loss of coronal tooth structure and lack of interocclusal space^20^.

There are no published data on the evaluation of surface roughness after mechanical load for performed pediatric crowns and lithium disilicate endo-crowns therefore, this laboratory study aimed to qualitatively evaluate and compare the surface roughness of stainless-steel crowns, zirconia crowns, and fiberglass crowns versus lithium disilicate endo-crowns as a restoration for pulpotomized primary molars. The null hypothesis (H0) of this study assumed that there was no difference between the mean surface roughness of performed pediatric crowns and lithium disilicate endo-crowns as a restoration of the primary molars.

## Material and methods

### Study setting and ethical consideration

An in-vitro controlled study was conducted at the Pediatric Dentistry Department, Faculty of Dentistry, Suez Canal University after receiving the Research Ethics Committee (REC) approval, Faculty of Dentistry, Suez Canal University (Code 387/2021) in compliance with the 1964 Helsinki Statement and its ensuing amendments. Parents provided informed written consent for the use of their children's extracted teeth in this study.

### Sample size calculation and randomization

G*Power (version 3.1.9.2, University Kiel, Germany. Copyright (c) 1992–2014)^21^ was used to calculate the sample size with 0.05 alpha (α) level and 0.05 Beta (β) level, i.e., power = 80%, effect size (f) = 0.31 based on a mean difference of sample size calculation of previous studies. The estimated sample size (n) should be 120 samples which were divided equally and randomly allocated into four groups (30 molar/group) according to the crown type.

The Research Randomizer software program was used to produce simple randomization (https://www.randomizer.org/)^22^. To guarantee concealed allocation into the four groups, an independent person placed randomization codes in serially numbered, opaque, secured envelopes.

### Eligibility criteria

Sixty recently extracted and/ or exfoliated mandibular primary first molars were collected from the outpatient clinic of the Pediatric Dentistry Department at Suez Canal University's Faculty of Dentistry. The selection criteria for chosen molars were having at least three axial walls of intact enamel crown margins, with at least 1 mm of sound tooth structure remaining and a minimum of one-third of the root was still unbroken with the floor. In addition, other sixty primary maxillary first molars, with sound occlusal surfaces, were selected for measurement of surface wear on antagonistic lower molar restorations while, molars having worn-out, too sharp, or fractured cusps were excluded.

### Teeth grouping

- The sixty primary mandibular first molars were randomly divided into four groups (15 molar/group) according to the crown type into group-ST, group-ZR, group-FG, and group-LD which were subjected to surface roughness evaluation after mechanical wear (Table 1).
- Other sixty sound, unprepared primary maxillary first molars were used for surface roughness evaluation after mechanical wear opposing the tested mandibular crowns.

**Table 1:** The composition and manufacturers of materials utilized in this study.

Materials	Composition	Manufacturers
Stainless steel crowns	Stainless steel alloy (8% nickel–chromium > 11%—molybdenum) crown	3M™ ESPE, USA
Zirconia crowns	Zirconium dioxide ZrO_2_	Kids-e-dental, LLP, India
Fiberglass crowns	aramid carbon or quartz mesh sheets incorporated into the dental resin	Figaro Crowns INC, USA
Lithium disilicate endo-crowns	Lithium disilicate Li_2_O_5_Si_2,_, IPS e.max Press/ Ivoclar Vivadent	Crown & Bridge Department, Faculty of Dentistry Suez Canal University

### Tooth specimen preparation

One hundred twenty eligible primary molars were wiped of blood, debris, and soft tissue after that disinfection was done using 0.1% thymol solution^23^. For a maximum of one month, the teeth samples were preserved at 37 °C in weekly replaced deionized water (SIGALD, Sigma-Aldrich Chemie GmbH)^24^. Then, each molar specimen was vertically embedded from the root portion up to 1 mm below the cementoenamel junction in a standard mold of 12 × 12 mm acrylic resin (Acrostone, Egypt)^25^.

### Pulpotomy procedure

Sixty mandibular primary molar specimens were prepared by the same operator. Firstly, No. 4 round bur was used on a high-speed handpiece with profuse water cooling for removing the enamel and dentin layer as well as superficial caries. Then, access opening was done with a No.330 carbide bur (Mani Inc., Japan). Root canal orifices were sealed with a thick mix of zinc oxide eugenol (ZOE) (PREVEST DenPro Limited, India) and then, covered by a glass ionomer cement layer (Riva-self cured SDI, Australia) in the lithium disilicate endo-crown group (group-LD) while the pulp chamber was completely packed with GIC filling material in other groups.

### Crown preparation and cementation

#### Preparation and cementation of SSCs and fiberglass crowns

In group-ST and -FG, 1.5 mm occlusal reduction was carried out with a flame-shaped diamond bur (Mani Inc., Japan). Mesial and distal surfaces were also reduced by about 1 mm to safeguard the snug fit stated by the manufacturer^26^. Then, appropriately sized preformed SSCs and Fiberglass crowns were selected and cemented with a glass ionomer cement (Riva-self-cured SDI, Australia).

#### Preparation and cementation of zirconia crowns

In group-ZR, orientation depth grooves were cut to guide the occlusal, facial, lingual, and interproximal reductions (0.5–1 mm for facio-lingual surfaces, 2 mm for occlusal reduction, and 1–2 mm for interproximal reduction)^27^. All surfaces were polished using a feather-edge stone permitting the zirconia crown’s passive fit. Subsequently, a suitable preformed zirconia crown was selected which was finally cemented with a resin cement (3M™ RelyX™ Universal Resin Cement, USA).

#### Preparation and cementation of lithium disilicate endo-crowns

In group-LD, each molar was prepared in two steps. Firstly, the occlusal preparation was carried out by doing 1.5 mm depth cuts with a tapered stone (TR-12 Dia-Bur, Mani), followed by reduction of the occlusal surface using a wheel stone (WR-13 Dia-Bur, Mani)^1^. The bur was positioned parallel to the occlusal plane to guarantee a flat surface, which establishes the location of the “cervical sidewalk” or “butt joint” finish line^28^. Secondly, the axial walls were flared utilizing a tapered stone (TR-12 Dia Bur Mani) at an eight-degree angle. The stone almost touched the glass ionomer base without excessive pressure. The same diamond bur was used to smooth the internal marginal angles then polishing with an abrasive rubber tip was done^28^.

A CAD-CAM scanner was used for scanning the lithium disilicate endo-crown specimens. After that, the endo-crown wax pattern was ground with a 1.5 mm standard occlusal thickness and then attached to a wax sprue before investing. For wax elimination, a preheating cycle was applied for one hour at 850 °C^1^ after that, the molds were set in an oven and pressed for 20 min with an IPS E.max Press ingot material (Ivoclar-Vivadent AG) at 915 °C. Subsequently, the endo-crown restorations were separated then finishing and glazing were done^1^.

For the cementation of lithium disilicate endo-crowns, the teeth specimens were acid-etched with a 37% phosphoric acid gel (T-Etchant, Nexobio, Korea) for 20 s, then thorough rinsing and air-dryness were done. Lithium disilicate endo-crown fitting surfaces were etched using a porcelain etchant of 9.5% hydrofluoric acid (9.5% HF acid, Bisco, USA) for 20 s, then thorough rinsing for 60 s followed by air-dryness. After that, a micro-brush was used to apply porcelain silane (Pentron, USA) and it was allowed to dry for 60 s. Finally, the self-adhesive resin cement (3 M™ RelyX™ Universal Resin Cement, USA) was placed on the fitting surface of the previously treated endo-crowns and the prepared molars using an auto-mix syringe. The lithium disilicate endo-crowns were positioned on their corresponding preparations using finger pressure. Each side was light-cured for 40 s after the excess cement was eliminated with a scaler^1,8^.

### Thermocycling

For one week, the primary molar specimens were preserved in distilled water at 37°C then subsequently exposed to 500 thermal cycles in the thermocycling device involving two water basins of 5° and 55 °C with a dwell time of 30 s^29^.

### Qualitative measurement of surface roughness

Two-body wear test was achieved with a programmable ROBOTA device conducted to four stations (chambers) multimodal chewing simulator (model ach09075dc-t, AdTech technology co., Germany). The sixty restored mandibular primary molar specimens were secured to the lower sample holder, while the other natural maxillary primary first molars, were fixed to the upper sample holder to imitate the primary occlusion. Each test specimen was subjected to a 5 kg weight, which corresponds to 50 N of chewing force^30^. The entire procedure was repeated for 250,000 cycles, which equates to approximately one year of artificial aging with artificial saliva serving as the chewing media^26,31^. The experimental conditions used in this study were 3mm vertical movement, 2mm horizontal movement, 55 mm/s rising speed, 55 mm/s descending speed, 55 mm/s forward speed, 55 mm/s backward speed, 0.8 Hz cycle frequency, and 5 kg weight per specimen^30^. The load was applied together with thermocycling, which requires dipping in a cold/hot water basin with a temperature change of 5ºC/55ºC and a dwell time of 60 s^30^.

The qualitative surface roughness (Ra) of tested crown specimens and antagonist upper primary first molars were assessed via a white light interference microscope (ZYGO Maxim-GP 200, Boston, Middlefield CT, USA). The occluding surfaces of each tested crown specimen and its antagonist primary molar were scanned before and after mechanical load application, and the surface roughness was evaluated in a 3D surface analyzer system. All specimens were scanned at a fixed magnification of 120X with a USB digital microscope equipped with an integrated camera (scope capture Digital Microscope, Guangdong, China) linked to an IBM-compatible computer. The images were captured with a resolution of 1280 × 1024 pixels. To standardize the roughness area measurement, Microsoft Office Manager used to crop the digital microscope images to 350 × 400 pixels. Then, WSxM software (Ver 5 develop 4.1, Nanotec, Electronica, SL) was used to analyze the cropped images to assess height averages (Ra) recorded in μm that were considered reliable indicators of surface roughness^32^. Then, a 3D image analysis software (Image J 1.43U, National Institute of Health, USA) was utilized, where the intact surfaces acted as a reference.

### Statistical analysis

All data was gathered, arranged, and statistically evaluated. The data was presented in the form of Mean ± Standard deviation (SD) values. One-way ANOVAs were used to compare the four study groups. Tukey's post-hoc test was performed for the evaluation of statistical significance among the groups and a paired sample t-test was used to assess the difference between groups. Values at p ≤ 0.05 were adopted as statistically significant. Statistical analysis was carried out using the SPSS software version 26 (Statistical Package for Social Science, Armonk, NY: IBM Corp).

### Ethical approval and consent to participate

Ethical approval for this study was obtained from the ethical committee (REC), Faculty of Dentistry, Suez Canal University (Code 387/2021) following the ethical guidelines outlined in the 1964 Helsinki Declaration and its subsequent revisions. Informed written consent from parents was attained to use their children’s extracted teeth in the research.

## Results

### Surface roughness (Ra) after mechanical wear of different crown types

**Figure 1:**
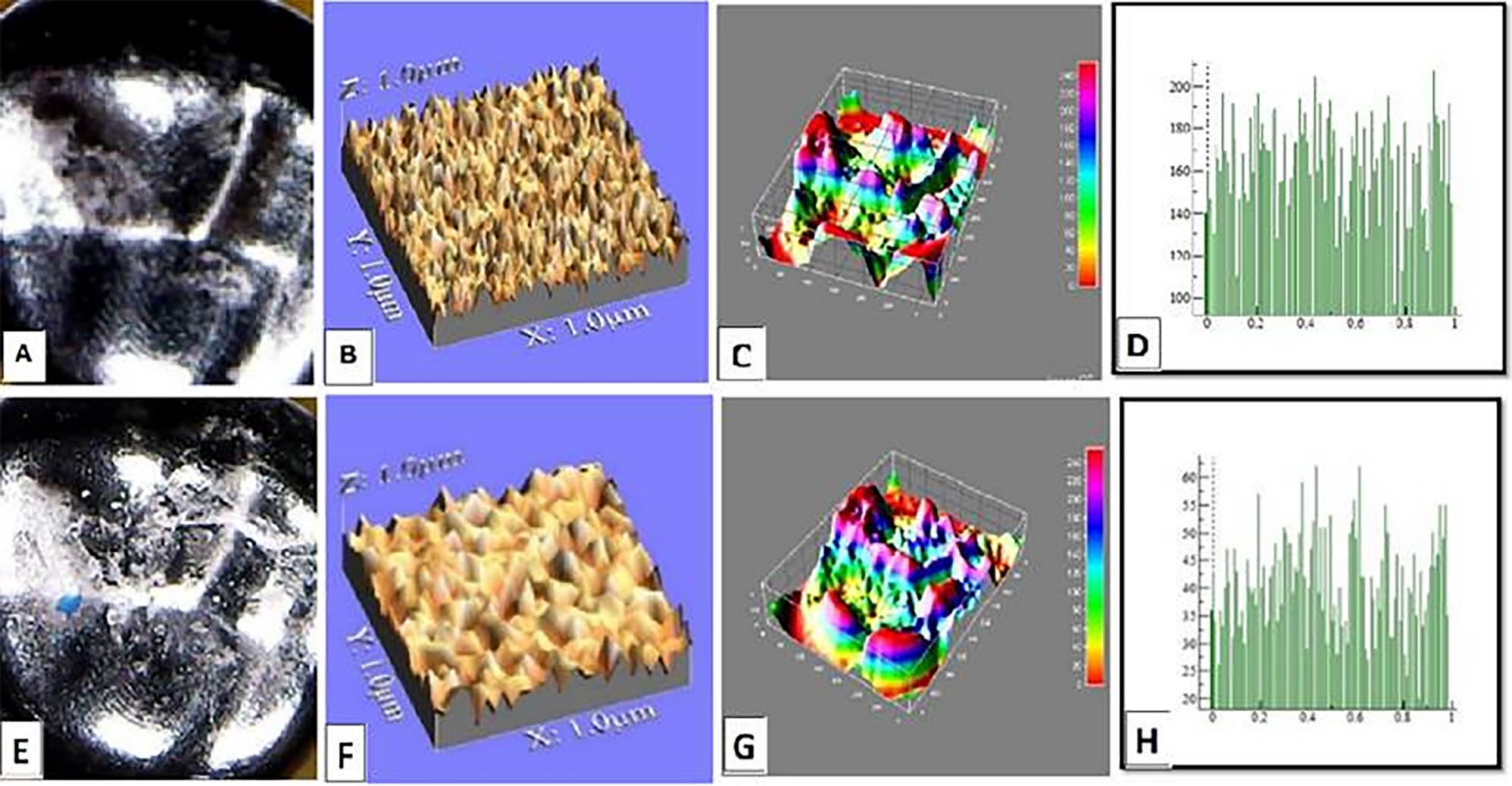
Stainless-steel crown: before mechanical load 3-D topographic change in crown surface, surface plot of 3-dimensional geometry of the worn surfaces and histogram showing surface roughness (**A**–**D**). After mechanical load 3-D topographic change in crown surface, surface plot of 3-dimensional geometry of the worn surfaces and histogram showing surface roughness (**E**–**H**).

**Figure 2:**
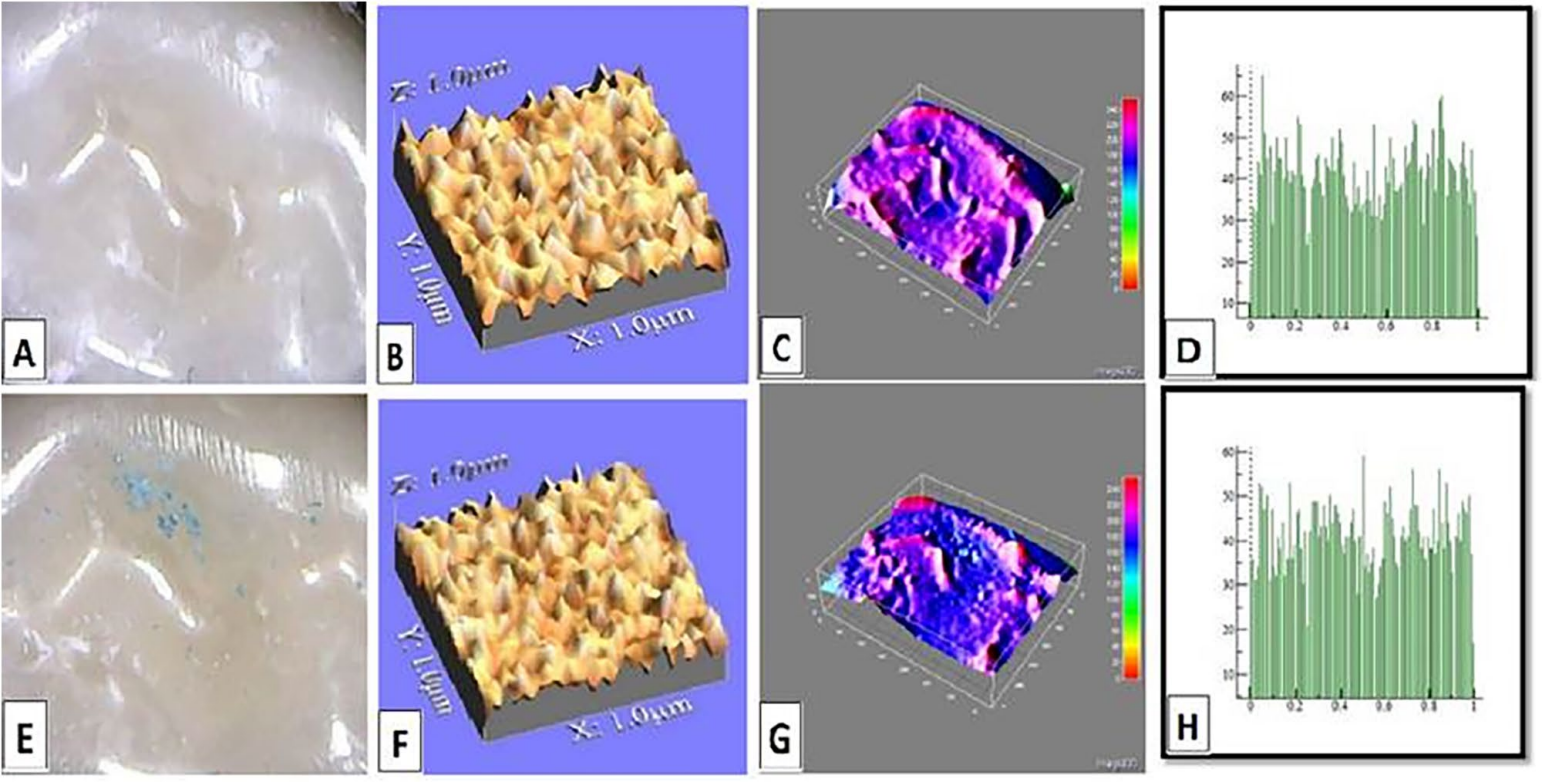
Zirconia crown: before mechanical load 3-D topographic change in crown surface, surface plot of 3-dimensional geometry of the worn surfaces and histogram showing surface roughness (**A**–**D**). After mechanical load 3-D topographic change in crown surface, surface plot of 3-dimensional geometry of the worn surfaces and histogram showing surface roughness (**E**–**H**).

**Figure 3:**
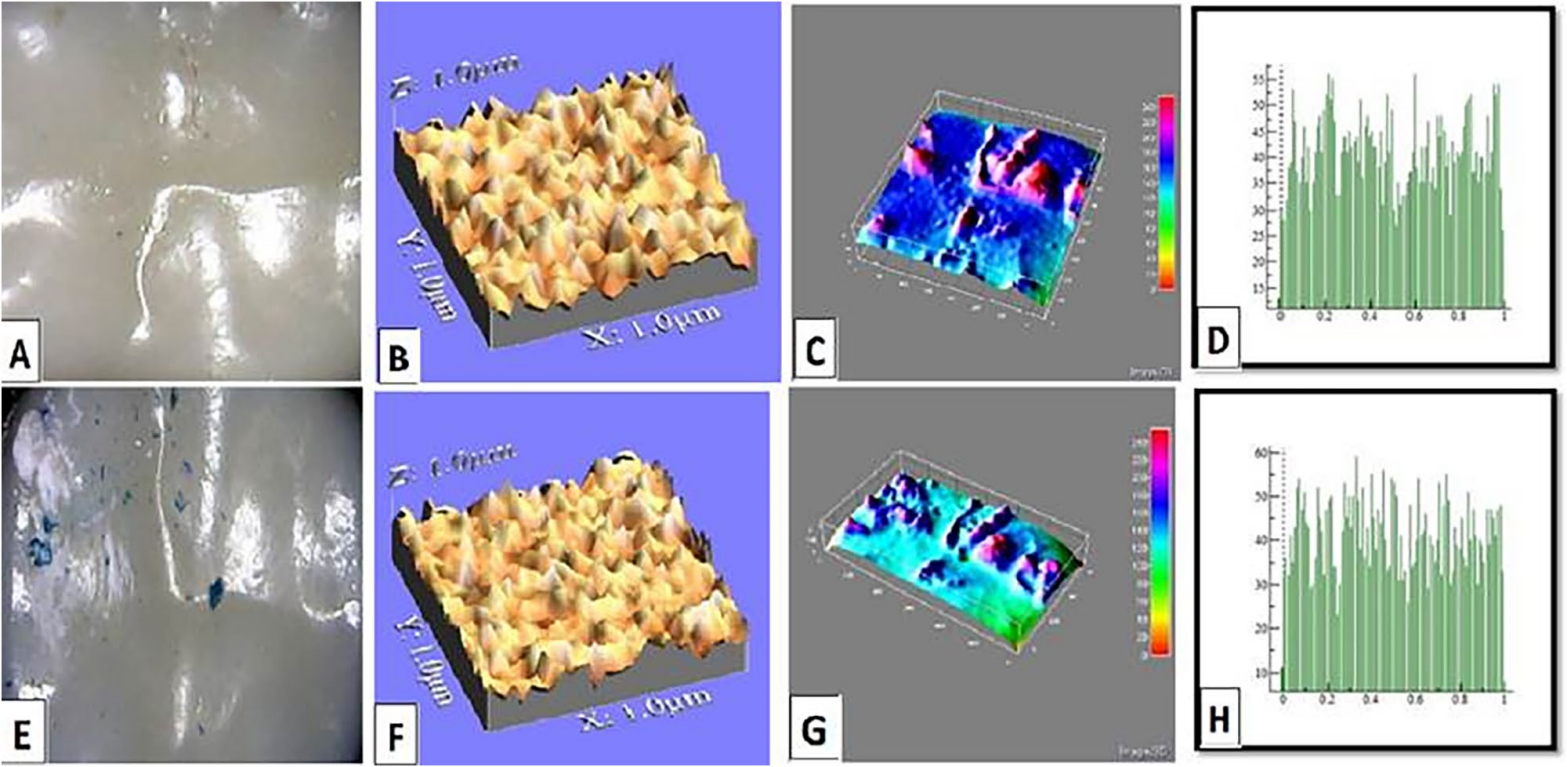
Fiberglass crown: before mechanical load 3-D topographic change in crown surface, surface plot of 3-dimensional geometry of the worn surfaces and histogram showing surface roughness (**A**–**D**). After mechanical load 3-D topographic change in crown surface, surface plot of 3-dimensional geometry of the worn surfaces and histogram showing surface roughness (**E**–**H**).

**Figure 4:**
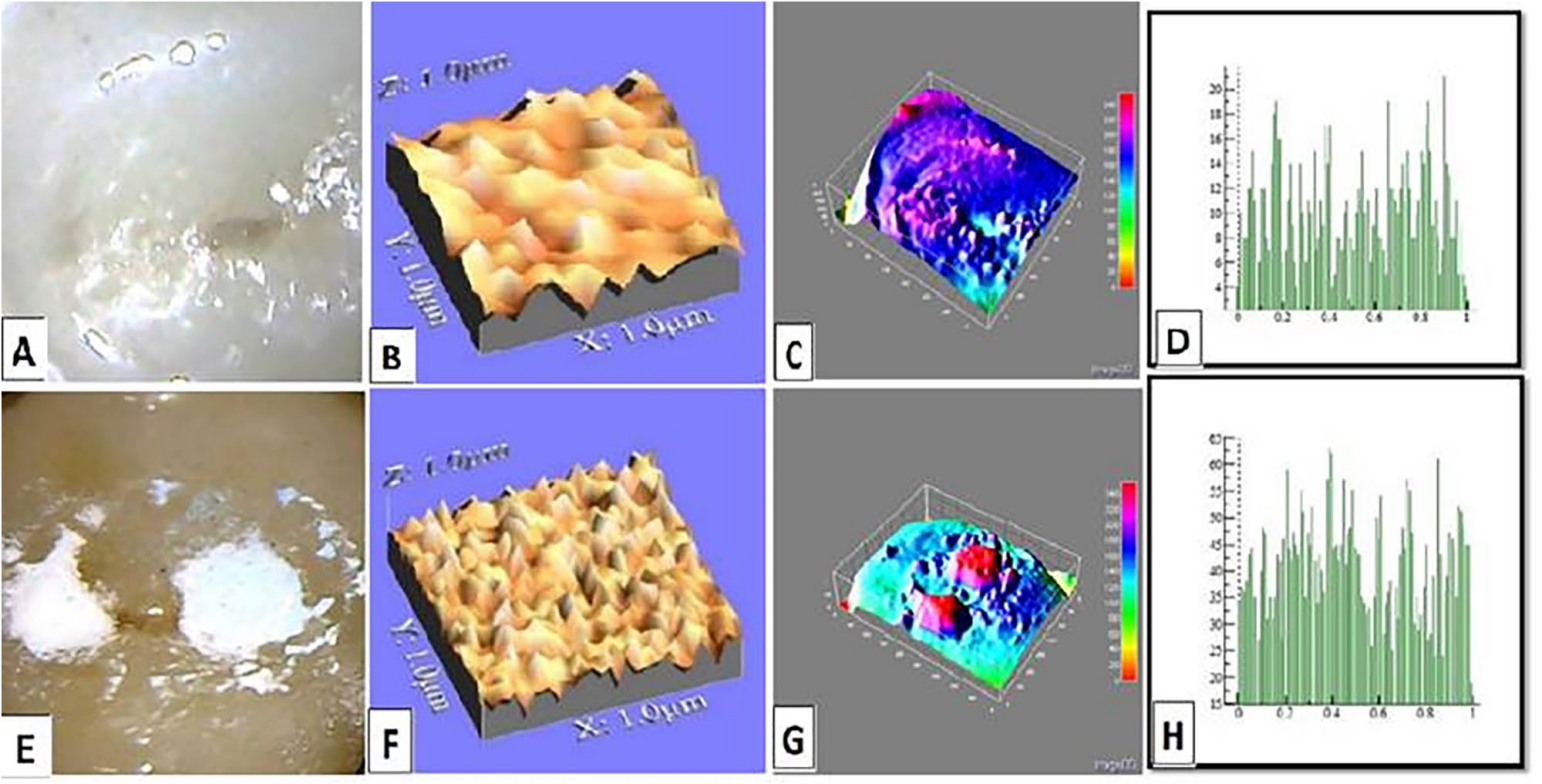
Endo-crown: before mechanical load 3-D topographic change in crown surface, surface plot of 3-dimensional geometry of the worn surfaces and histogram showing surface roughness (**A**–**D**). After mechanical load 3-D topographic change in crown surface, surface plot of 3-dimensional geometry of the worn surfaces and histogram showing surface roughness (**E**–**H**).

The mean values of surface roughness (μm) for the four tested crowns before and after mechanical wear, are presented in Table 2. All tested crowns showed an increase in surface roughness after mechanical wear with no statistically significant difference except for Fiberglass crowns which had the highest change in surface roughness with a statistically significant difference (p = 0.010) (Figs. 1, 2, 3, 4).

**Table 2:** Comparison of surface roughness of different crown types.

Groups	Ra (μm) (Mean ± SD)		Paired- t-test	p-value^†^
	Before	After		
Group-ST	0.2448 ± 0.005^cA^	0.2481 ± 0.005^bA^	1.24	0.209
Group-ZR	0.2567 ± 0.01^aA^	0.2574 ± 0.001^aA^	1.53	0.681
Group-FG	0.2499 ± 0.005^bB^	0.2575 ± 0.002^aA^	3.16	0.010*
Group-LD	0.2506 ± 0.001^bA^	0.2518 ± 0.003^bA^	1.03	0.290
F-test	7.75	7.61		
P-value	0.001*	0.001*		

*, and Different small superscript letters in the same column indicate statistically significant differences using one-way ANOVA (p ≤ 0.05).

^†^Different capital superscript letters in the same row indicate statistically significant differences using pairs sample T-Test (p ≤ 0.05).

### The surface roughness (Ra) after mechanical wear of antagonist primary molars to different crown types

**Figure 5:**
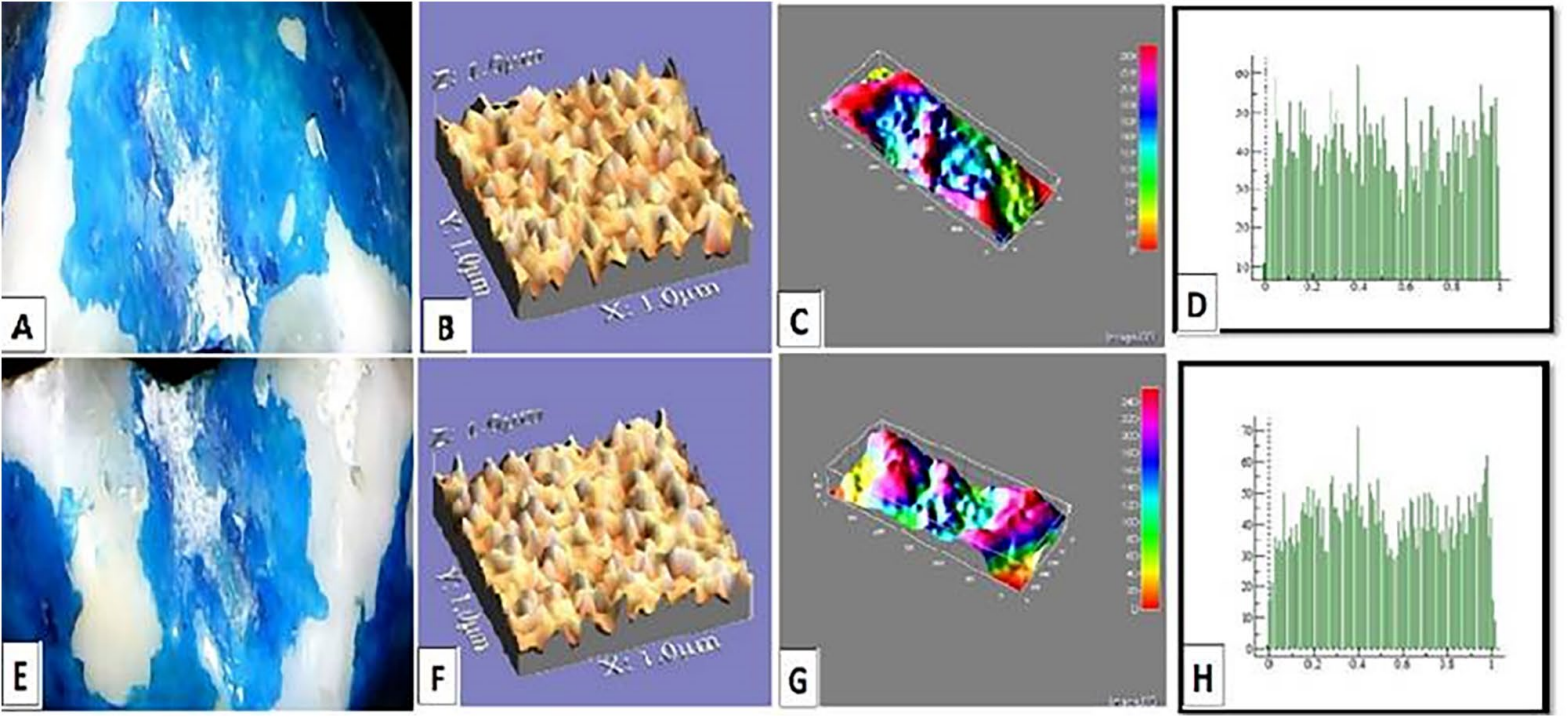
Antagonist to Stainless-steel crown: before mechanical load 3-D topographic change in occlusal surface, surface plot of 3-dimensional geometry of the worn surfaces and histogram showing surface roughness (**A**–**D**). After mechanical load 3-D topographic change in occlusal surface, surface plot of 3-dimensional geometry of the worn surfaces and histogram showing surface roughness (**E**–**H**).

**Figure 6:**
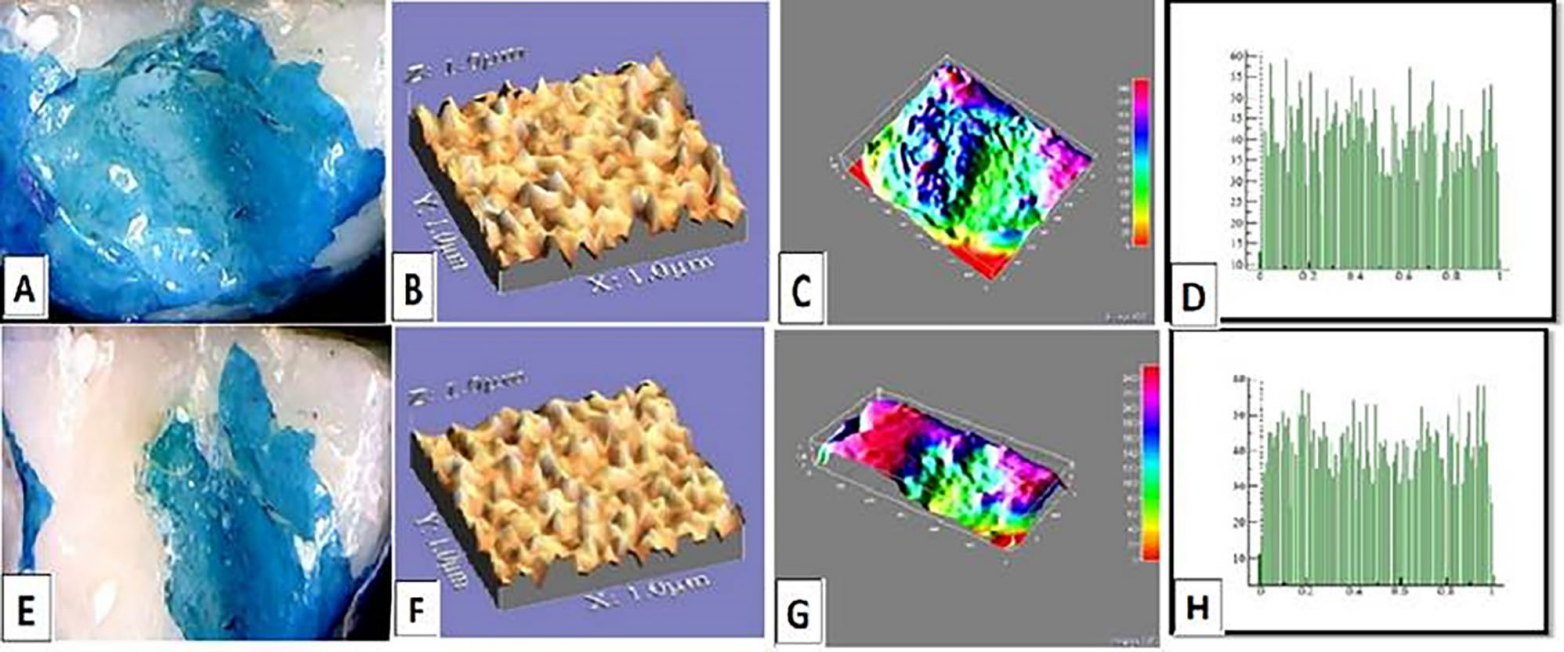
Antagonist to Zirconia crown: before mechanical load 3-D topographic change in occlusal surface, surface plot of 3-dimensional geometry of the worn surfaces and histogram showing surface roughness (**A**–**D**). After mechanical load 3-D topographic change in occlusal surface, surface plot of 3-dimensional geometry of the worn surfaces and histogram showing surface roughness (**E**–**H**).

**Figure 7:**
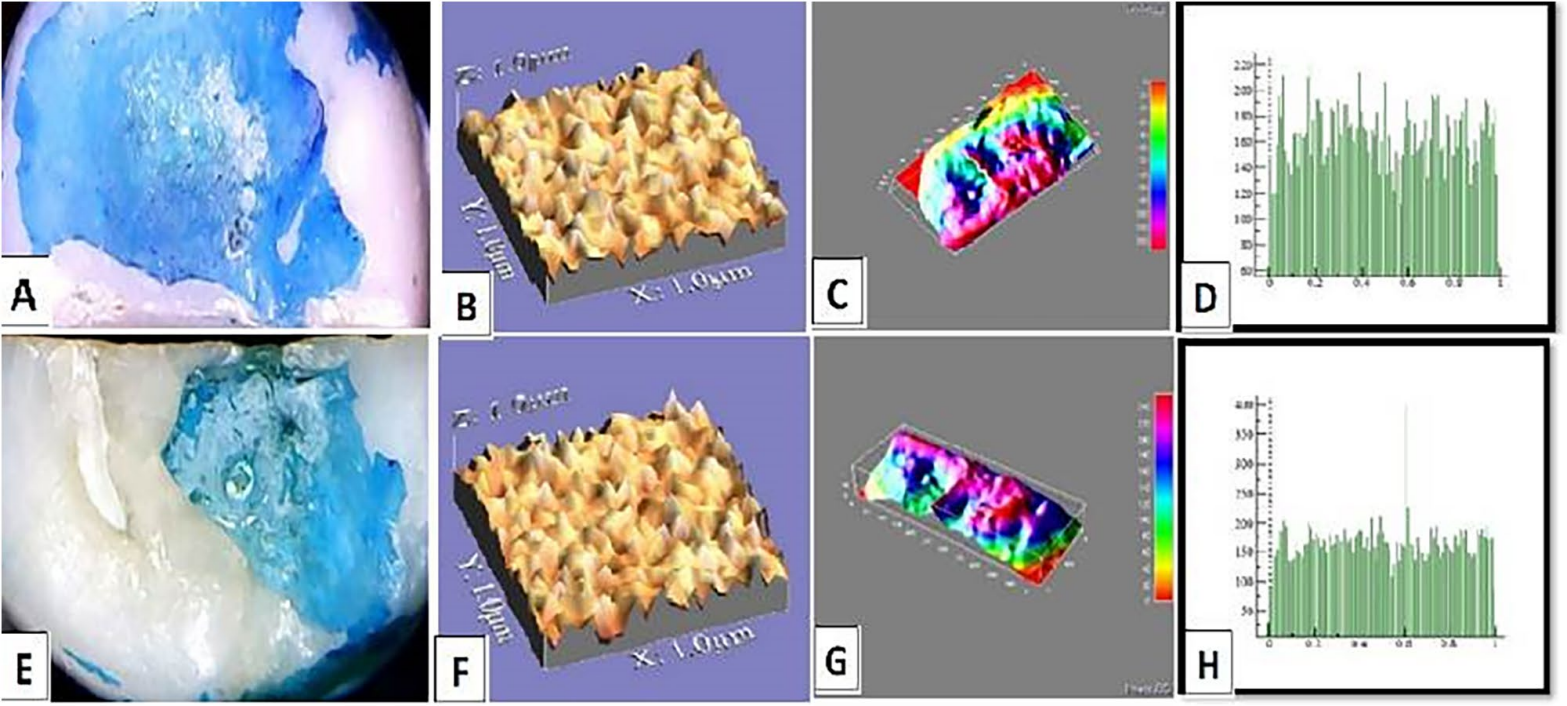
Antagonist to Fiberglass crown: before mechanical load 3-D topographic change in occlusal surface, surface plot of 3-dimensional geometry of the worn surfaces and histogram showing surface roughness (**A**–**D**). After mechanical load 3-D topographic change in occlusal surface, surface plot of 3-dimensional geometry of the worn surfaces and histogram showing surface roughness (**E**–**H**).

**Figure 8:**
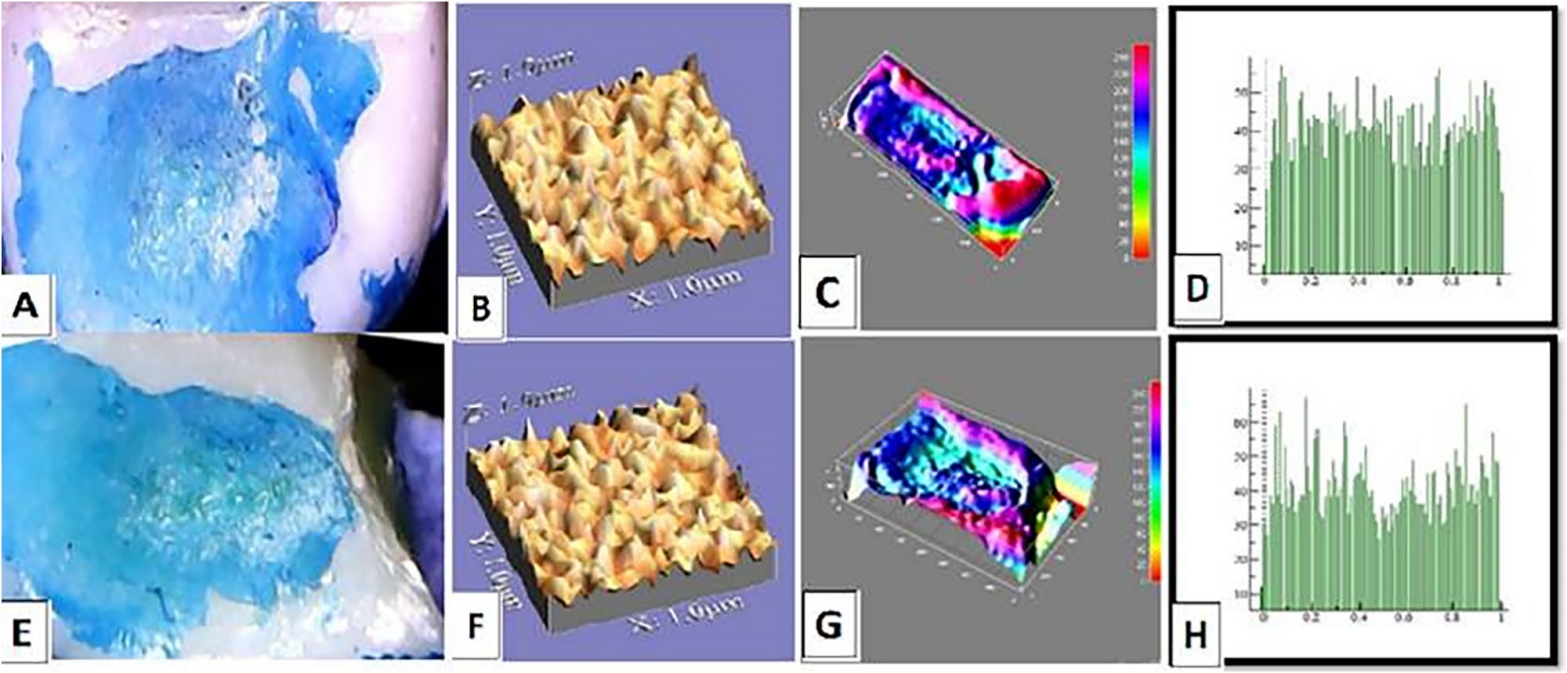
Antagonist to Endo-crown: before mechanical load 3-D topographic change in occlusal surface, surface plot of 3-dimensional geometry of the worn surfaces and histogram showing surface roughness (**A**–**D**). After mechanical load 3-D topographic change in occlusal surface, surface plot of 3-dimensional geometry of the worn surfaces and histogram showing surface roughness (**E**–**H**).

The mean surface roughness values of antagonist upper primary first molars opposing SSC crowns were 0.2499 ± 0.001 and 0.2501 ± 0.001 μm before and after mechanical wear respectively with non-statistically significant differences (p = 0.725) (Table 3; Fig. 5). On the other hand, a statistically significant difference was found between the mean surface roughness values before and after mechanical wear of antagonist upper primary first molars opposing to zirconia, fiberglass, and lithium disilicate endo-crowns (p < 0.001) (Figs. 6, 7, 8). The surface roughness values in antagonist upper primary first molars opposing SSCs revealed the lowest amount of tooth wear (0.0002 μm). Statistically significant differences between the mean surface roughness values of antagonist upper primary first molars opposing to tested crowns after mechanical wear were observed (p < 0.001).

**Table 3:** Comparison of surface roughness of antagonist primary maxillary molars opposing different crown types.

Groups	Ra (μm) (Mean ± SD)		Paired- t-test	p-value^†^
	Before	After		
Antagonist to group-ST	0.2499 ± 0.001^bA^	0.2501 ± 0.001^bA^	0.373	0.725
Antagonist to group-ZR	0.2478 ± 0.001^cB^	0.2567 ± 0.001^aA^	6.21	≤ 0.001*
Antagonist to group-FG	0.2470 ± 0.001^bB^	0.2493 ± 0.001^bA^	6.429	≤ 0.001*
Antagonist to group-LD	0.2519 ± 0.001^aB^	0.2570 ± 0.001^aA^	8.146	≤ 0.001*
F-test	10.19	8.284		
P-value	0.001*	0.001*		

*, and Different small superscript letters in the same column indicate statistically significant differences using one-way ANOVA (p ≤ 0.05).

^†^Different capital superscript letters in the same row indicate statistically significant differences using pairs sample T-Test (p ≤ 0.05).

## Discussion

Rehabilitation of pulpotomized primary molars with extensive coronal damage remains a clinical challenge since the removal of pulp as well as neighboring dentin is linked with coronal weakness^33^. Stainless steel crowns have been regarded as the gold standard for restoring pulpotomized primary molars. Conversely, with the development of Esthetic dentistry, both children and their caretakers have been looking for more appealing alternatives excluding stainless steel crowns^34^.

CAD/CAM technology is an advanced method of producing indirect restorations in primary teeth. With the aid of the CAD/CAM software design, the dentist can generate perfect occlusal and proximal contact points and an improved marginal fitting at the gingival wall^35^. So, in this study, endo-crowns were selected for restoration of pulpotomized primary molar as it is conservative with a smaller clinical chairside time and less wear of the opposing teeth. Lithium disilicate ceramic was selected to be the material of choice for the construction of endo-crowns in the current study because it guarantees the mechanical strength required for withstanding the occlusal forces applied on the tooth, in addition to the restoration's bond strength to the cavity walls^36^.

The inclusion or lack of fillers, the type, quantity, and size of the nanofillers, as well as how the filler particles are incorporated into the resin matrix, can all affect the wear resistance of restoration^37^. With the growing evolution of new aesthetic full coverages for primary teeth, there is a higher need for assessing the resulting pathological tooth wear with these types^38^. So, this study aimed to evaluate the surface roughness changes in SSCs, zirconia crowns, Fiberglass, and lithium disilicate endo-crowns also, evaluating the opposing primary enamel wear. Human teeth were chosen for this research because the contour of the pulp chamber and root canals provide a more precise crown-to-root ratio compared to artificial teeth^1^.

According to the present study results, the null hypothesis was rejected since there were significant differences in surface roughness among the tested groups. Zirconia crowns in this study had a low change in surface roughness after mechanical wear which may be probably attributed to its superior physical properties that allow the maintenance of a smooth surface and prevent surface microfractures during the wear test, this agreed with Miyazaki et al.^39^ who stated that zirconia crowns demonstrated minor wear, as a result of their fine uniform structure, despite their high hardness. Also, in the current study, Fiber-glass crowns revealed the highest change in surface roughness after mechanical wear among the tested groups with a statistically significant difference (p = 0.01). This agreed with El Habashy and Aboushelib.,^12^ who reported that Figaro crowns (0.88 ± 0.2 mm3) had a meaningly higher amount of 3-D wear in comparison to stainless steel crowns (0.09 ± 0.02 mm3) (P < 0.01). Moreover, this study finding was in line with Ohlmann et al.^40^ who concluded that the total mean wear of posterior polymer crowns with and without a glass‐fiber framework was substantially greater than for metal‐ceramic crowns within a 12‐month follow-up period. In addition, the lithium disilicate endo-crown had less change in surface roughness which may be due to the butt plane and retainer design that are deeply settled into the internal portion of the pulp chamber and the cavity margins^19^.

According to this study's results, zirconia crowns caused antagonist enamel wear that was higher than that caused by other tested crowns; this agreed with Walia et al.^41^ who proved increased wear of natural antagonists to zirconia crowns. Also, Contreras et al.^42^ linked the noticed surface wear of the antagonists opposing zirconia and endo-crown restorations to the non-homogeneity of the glaze material and the presence of gaps in the form of “islands” in the ceramic surface which resulted in numerous irregularities and glaze accumulation. On the other hand, this disagreed with Bolaca and Erdoğan.^43^ who observed that zirconia caused lesser antagonist wear than did the other ceramics. Also, Murali et al.^44^, denied the presence of any wear on natural teeth opposing zirconia crowns.

Our results recorded the higher surface roughness in the surface enamel of antagonist primary teeth opposed to zirconia compared to fiberglass crown; this agreed with Peng et al.^45^ who found that zirconia crowns were the most abrasive to their antagonists compared to nanohybrid and stainless-steel crowns (P < 0.001). Also, these results coincided with Talekar et al.^46^, who found that the wear of the antagonistic tooth is more noticeable with zirconia than with glass fiber-reinforced composite crowns (GFRC). This could be credited to the different hardness of these materials, as the microhardness of GFRC (29.35 ± 2 HV) was considerably less than that of primary enamel (105 ± 4 HV), whereas the microhardness of the zirconia crown (1157 ± 7 HV) was ten times greater than that of primary enamel^46^.

Also, the SSCs group showed the least tooth loss in the antagonist upper primary first molars in this study; this agreed with Choi et al.^30^ who revealed that antagonist tooth volume losses in the Leucite and lithium groups were substantially higher than those in the steel group (P < 0.05). This may be linked with the ductility of the steel which absorbs the occlusal forces. Furthermore, metal crowns have a relatively smooth surface, that decreases the material abrasiveness. In this study, the high surface roughness of opposing enamel to lithium disilicate endo-crown group may be clarified by the development of wear debris as the glass particles liberated by wear act as abrasives, a phenomenon known as the “three-body wear mechanism”^47^.

This in-vitro study has limitations in that the forces generated intraorally vary in their magnitude, speed of application, and direction while the present research presented a controlled environment for comparing the material behavior beneath unchanged direction and speed conditions. In addition, two-body wear was evaluated in this study, and three-body wear tests could result in different outcomes. Therefore, a long-term clinical evaluation is needed to precisely assess the impact of various crown coverage materials on the structure of primary enamel.

## Conclusion

Based on the present study results, it was concluded that:

- Zirconia crowns had the least change in surface roughness after mechanical wear compared to other groups while causing the highest antagonist enamel wear than that caused by other tested crowns.
- The fiberglass crown had the highest change in surface roughness after mechanical wear which may indicate its low clinical endurance.
- Preformed SSCs showed the least tooth loss in the antagonist enamel compared to other groups.
- Lithium disilicate endo-crowns had comparable results to preformed SSCs and zirconia crowns providing a promising, conservative, and esthetic treatment.

### Clinical relevance

Enamel surface wear is a normal process that can be sped up by placing restorations whose wear characteristics are different from the natural tooth structure. In addition to causing aesthetic impairment, excessive enamel wear may cause clinical problems such as dentine hypersensitivity, temporomandibular joint remodeling-related poor masticatory function, damage to teeth's occluding surfaces, loss of vertical dimension of occlusion, and tooth death. It is recommended to use lithium disilicate endo-crown in pediatric clinical practice due to its comparable less antagonist enamel loss along with offering a conservative, esthetic restoration with minimal change in surface roughness.

## Data Availability

On reasonable request, the datasets utilized and/or analyzed during the present study are accessible from the corresponding author.
